# Mitochondrial proteomics on human fibroblasts for identification of metabolic imbalance and cellular stress

**DOI:** 10.1186/1477-5956-7-20

**Published:** 2009-05-28

**Authors:** Johan Palmfeldt, Søren Vang, Vibeke Stenbroen, Christina B Pedersen, Jane H Christensen, Peter Bross, Niels Gregersen

**Affiliations:** 1 Research Unit for Molecular Medicine, Institute of Clinical Medicine, Aarhus University Hospital and Faculty of Health Sciences, University of Aarhus, Brendstrupgaardsvej 100, 8200 Aarhus N, Denmark

## Abstract

**Background:**

Mitochondrial proteins are central to various metabolic activities and are key regulators of apoptosis. Disturbance of mitochondrial proteins is therefore often associated with disease. Large scale protein data are required to capture the mitochondrial protein levels and mass spectrometry based proteomics is suitable for generating such data. To study the relative quantities of mitochondrial proteins in cells from cultivated human skin fibroblasts we applied a proteomic method based on nanoLC-MS/MS analysis of iTRAQ-labeled peptides.

**Results:**

When fibroblast cultures were exposed to mild metabolic stress – by cultivation in galactose medium- the amount of mitochondria appeared to be maintained whereas the levels of individual proteins were altered. Proteins of respiratory chain complex I and IV were increased together with NAD^+^-dependent isocitrate dehydrogenase of the citric acid cycle illustrating cellular strategies to cope with altered energy metabolism. Furthermore, quantitative protein data, with a median standard error below 6%, were obtained for the following mitochondrial pathways: fatty acid oxidation, citric acid cycle, respiratory chain, antioxidant systems, amino acid metabolism, mitochondrial translation, protein quality control, mitochondrial morphology and apoptosis.

**Conclusion:**

The robust analytical platform in combination with a well-defined compendium of mitochondrial proteins allowed quantification of single proteins as well as mapping of entire pathways. This enabled characterization of the interplay between metabolism and stress response in human cells exposed to mild stress.

## Background

Mitochondrial activity is essential for human health and the number of disorders known to be related to mitochondrial dysfunction is increasing. Defects in mitochondrial functionality cause a wide range of diseases [1], including respiratory chain defects [2], fatty acid oxidation deficiencies [3] and neurodegenerative diseases [4]. Mitochondrial proteins are important regulators of apoptosis and mitochondrial dysfunction is an important factor in aging, diabetes mellitus, cancer [5,6], cardiovascular disease [7,8] as well as Alzheimer's and Parkinson's disease [9]. Monogenetically inherited mitochondrial defects are commonly detected indirectly from metabolite signatures in blood or urine, or by sequencing of candidate genes. For more specific assessment of mitochondrial activity, enzymatic activity can be measured on mitochondria isolated from cultivated patient fibroblasts from which selected mitochondrial protein markers also can be quantified using immunological detection. However, a proteomic survey might be advantageous in the investigation of complex defects where several factors may contribute to disease. Relative quantification of proteins from mitochondrial pathways would enable the detection of imbalanced metabolism and stress in mitochondria, serve as a starting point for the selection of disease marker proteins and be used for exploration of disease etiology. The human mitochondrial proteome has been computationally predicted to contain proteins from at least a thousand different genes [10,11].

Experimental approaches to define the mitochondrial proteome using mitochondria purified with gradient centrifugation have yielded more than six hundred mitochondria-associated proteins from human mitochondria [12,13] and even higher numbers in studies on mouse mitochondria [11,14]. These studies have mainly been discovery studies targeted to identify as many proteins as possible from a large amount of purified mitochondria. From a clinical perspective the proteomic methods should be applicable to a low amount of patient material and they should be quantitative. Reliable and fast quantification methods for mass spectrometry (MS) based proteomics are being developed and support the growing applications of proteomic techniques in research and diagnosis of genetic and metabolic disorders [15]. A suitable method for large scale analysis of relative protein quantities is mass spectrometry analysis of peptides chemically labeled by isobaric tags for relative and absolute quantification (iTRAQ) [16]. iTRAQ allows simultaneous determination of both identity and relative abundance of peptides in tandem mass spectra and has gained popularity due to its high sensitivity and robustness, and because it allows simultaneous measurement of 4–8 samples [17-20].

The present work establishes an iTRAQ-based proteomic method for relative quantification of mitochondrial proteins in human fibroblasts. In order to obtain comprehensive proteome data we manually assembled and curated a compendium containing proteins from the mitochondrial metabolism and stress response systems. Our method was tested on human skin fibroblasts, which in many cases is the only tissue material readily available from patients. Proteomics of cultivated fibroblasts is suitable for studying the consequences of human diseases at the protein level, and cultivation in controlled environments enables studies conducted under stressful conditions. Characterization of cultivated fibroblasts with respiratory chain deficiencies has previously been performed by cultivation in galactose. Galactose cultivation results in altered energy metabolism – a mild metabolic stress- which enhances the effects of mitochondrial disorders [21,22]. In the current work we studied the mitochondrial proteome as a function of cultivation in galactose versus glucose medium and describe how wild type cells respond to the metabolic stress.

## Materials and methods

### Cell cultures

Primary normal human dermal fibroblasts (NHDF) from newborn males (Camprex *#*CC-2509 annotated NHDF-1 and ATCC #CRL-2429 annotated NHDF-2), were cultivated at 37°C and 5% (v/v) CO_2 _in RPMI 1640 medium (BioWhittaker) containing 10% (v/v) fetal calf serum (BioWhittaker). Cells for experiments were used between passage 9 and 13. After preculturing, the cultures were transferred to 150 cm^2 ^flasks and harvested at sub-confluence after approximately 72 h in RPMI 1640 medium (with 2 g/l glucose), or glucose-free RPMI 1640 medium supplemented with 2 g/l galactose, both supplemented with 10% fetal calf serum.

### Mitochondrial enrichment

Cells from four 150-cm^2 ^flasks were resuspended in 10 ml MOPS buffer (10 mM, pH 7.2) containing sucrose (200 mM), EDTA (0.1 mM) and protease inhibitor (Complete from Roche). The cells were disrupted on ice by 30 strokes in a Dounce homogenizer. Cell debris was removed by two centrifugation steps at 600 × g for 7 minutes, where the pellets were discarded and the resulting supernatant was centrifuged at 10,000 × g for 15 min. The pellet containing mitochondria was washed in the MOPS buffer (pH 7.2) without protease inhibitor, centrifuged at 10,000 × g for 15 min and stored at -80°C. After adding a sample buffer from the iTRAQ kit (Applied Biosystems, Foster City, California, USA) consisting of 0.5 M triethylammonium bicarbonate buffer (pH 8.5) with 0.1% SDS, the samples were treated with ultrasonication (Branson Sonifier 250, Branson Ultrasonics corp., Danbury, USA) at output control 3 and 30% duty cycle for three rounds of 10 seconds with one minute on ice between each round.

### Western blot analysis

The mitochondrial protein samples, 6 μg per well, were separated on a 12% SDS-bis-Tris polyacrylamide gel (BioRad). Each sample was loaded in triplicate, and a standard dilution series with five concentrations in duplicate was loaded on each gel for quantitative purposes. Blotting to a PVDF membrane was performed on a Semi-Dry Transfer Cell (BioRad). The detection procedure was according to instructions from the manufacturer of ECL Plus Western Blotting Detection Reagents (GE Healthcare). The membrane was incubated over night with primary antibodies against NDUFA9 (MitoSciences, Eugene, Oregon, USA) and VDAC1/porin (Abcam, Cambridge, Massachusetts, USA). The blots were scanned on ChemiDoc (UVP, Upland, California, USA) and densitometry was performed in ImageQuant 5.0 software (Molecular Dynamics, Sunnyvale, California, USA). The ratio between the protein amount of NDUFA9 and the loading control VDAC1 was calculated for each lane and the three resulting values from glucose and galactose samples were compared.

### iTRAQ labeling, IEF separation and purification of peptides

Protein concentrations in the samples enriched for mitochondria were measured by the Bradford assay (Bio-Rad Laboratories) and 100 μg of each protein sample was processed according to iTRAQ manufacturer's instruction (Applied Biosystems). Each protein sample was digested with 2 μg trypsin (Trypsin Gold from Promega, Madison, Wisconsin, USA) overnight at 30°C in iTRAQ sample buffer. Different combinations of the 4-plex iTRAQ labels, two labels per LC-MS/MS run, were used in the four different experiments, to minimize risks of systematic errors. After iTRAQ-labeling the peptide samples were combined and subsequently purified using a strong cation exchange (SCX)-cartridge; Strata from Phenomenex (Torrence, California, USA). Before loading, the samples were adjusted to pH 3.0 by dilution at least a factor ten in 10 mM phosphoric acid with 25% acetonitrile (AcN) and pH 3.0, which also served as washing buffer. The peptides were eluted with a mixture of 5% of ammonia and 30% methanol and subsequently vacuum-dried. The peptides were separated by isoelectric focusing (IEF) on a Multiphor II unit (Pharmacia Biotech AB, Uppsala, Sweden) using an Immobiline Drystrip Gradient (IPG) pH 3.5–4.5 gel (GE Healthcare, Uppsala, Sweden), a pH range previously shown to give high proteome coverage [23]. The sample was dissolved in rehydration solution, containing 8 M urea, 0.5% IPG buffer 3.5–5 (GE Healthcare) and 0.002% bromophenol blue, and the 18 cm Drystrip was rehydrated overnight. IEF was run for 59 kVh with the following program: 1 min gradient from 0–500 V, 1.5 h gradient from 500–3500 V followed by 16 h at 3500 V. The gel strip was wiped with filter paper to remove excess cover oil from IEF and cut in ten pieces of equal size. Peptides were extracted from the gel in two steps, of one hour each, with 100 μl 5% AcN, 0.5% trifluoracetic acid (TFA), and purified on PepClean C-18 Spin Columns (Pierce, Rockford, Illinois, USA) according to manufacturer's protocol.

### Nano-liquid chromatography and mass spectrometry (MS) analysis

The peptide mixtures were separated by liquid chromatography (Easy nLC from Proxeon, Odense, Denmark) coupled to mass spectrometry (LTQ-Orbitrap, Thermo Fisher Scientific, Waltham, USA) through a nano-electrospray source with stainless steel emitter (Proxeon). The peptides were separated on a reverse phase column, 75 μm in diameter and 100 mm long, packed with 3.5 μm Kromasil C18 particles (Eka Chemicals, Bohus, Sweden) at a flow of 300 nL/minute using a 100 minutes gradient of AcN in 0.4% acetic acid; starting with 5% and ending with 35% AcN. The mass spectrometry detection constituted of full scan (m/z 400–2000) with Orbitrap detection at resolution R = 60,000 (at m/z 400) followed by up to four data dependent MS/MS scans, with linear ion trap (LTQ) detection of the most intense ions. Dynamic exclusion of 25 s was employed as well as rejection of charge state +1 and real time recalibration [24] by lock mass on m/z 445.120025. Pulsed Q dissociation (PQD) fragmentation was performed with activation time of 0.1 s and activation Q of 0.7. For efficient fragmentation and detection of iTRAQ reporter ions, normalized collision energy of 33 was used since optimization experiments showed that it gave the highest number of identified peptides with iTRAQ signal. Selected ion monitoring (SIM) was designed as data dependent scanning targeting m/z values of proteotypic peptides (typically two peptides per protein), which had been identified in the previous experimental runs. SIM analyses were performed using full scan in LTQ, followed by SIM in Orbitrap (with a mass width window of ± 3 m/z units) and MS/MS in LTQ. Thus the fragmentation scans and acquisition of iTRAQ signal in the SIM analyses were performed in the same way as in the previous standard experimental runs. Approximately 35 peptides were on the inclusion list per run, with retention time limit of ± 5 minutes.

### Database searches and statistics

The raw data files were processed using extract_msn.exe (Thermo Fischer Scientific) to generate peak lists of the tandem spectra. The processed data was searched with Mascot  version 2.2.04 (Matrix Science, London, UK), which was used for protein identification and iTRAQ reporter quantification. Full scan tolerance was 5 ppm, MS/MS tolerance was 0.9 Da, and up to two missed cleavages were accepted. Fixed modifications were those originating from iTRAQ protocol: iTRAQ-4plex of lysine and N-terminal and methylthio modification of cysteines, whereas oxidation of methionine and iTRAQ-4plex of tyrosine were set as variable modifications. The threshold of significance was set to 0.001, which resulted in a false discovery frequency of less than 0.003 when searched in Mascot against the decoy database of random sequences. In each study, all generated peak lists, from standard analyses as well as from SIM analyses, of the ten different fractions of peptides were merged together. The merged files were searched against the IPI human database version 3.45 (71,983 sequences, released 6/10/2008) using the MudPIT scoring algorithm of Mascot. Protein identification data can be found in additional files [see Additional files 1, 2, 3 and 4]. Throughout the manuscript the HGNC symbol  obtained from the IPI-database was used to refer to protein hits. iTRAQ values were reported for proteins with three or more measured iTRAQ values, where each peptide should have an expectation value of 0.02 or below. iTRAQ quantitation was performed in Mascot, were normalization to summed intensities was applied to compensate for possible variation in starting material. For details see . Three iTRAQ-studies were performed comparing galactose and glucose cultivation of the fibroblast NHDF-1. The three studies were performed at different times and on independent cultivations. The iTRAQ-ratio of galactose to glucose values were calculated for each protein from the three independent studies giving independent triplicate values. Average galactose to glucose ratios for each protein was reported as significantly different from 1.0 if they passed two tests 1) a threshold test of two times the global standard error (2 × 0.055 = 0.11) and 2) a two-tailed student's T-test for equal variance data.

## Results

We have performed proteomic analyses on mitochondria from cultivated human skin fibroblasts to obtain an overview of the mitochondrial protein levels and thus detect stress response and unbalanced metabolism. The method, including all steps from cultivation to data analysis, was developed and standardized to obtain robust and easily interpretable data.

### Amount of mitochondrial proteins in galactose and glucose cultivations

**Figure 1:**
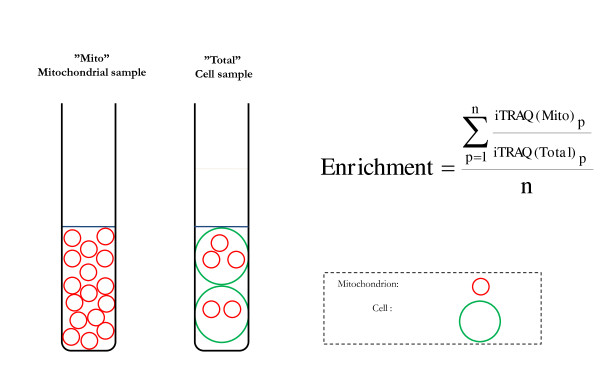
**Schematic picture describing the mitochondrial enrichment factor**. The mitochondrial enrichment factor was calculated as the average amount of 39 mitochondrial proteins (n = 39) in mitochondrial sample compared with the total cell sample. The enrichment factor represents an indirect measure of the relative amounts of mitochondrial proteins in the different fibroblast cultivations.

Cultivation in the slowly metabolized sugar galactose is a way of inflicting metabolic stress on the cell [21,25]. Fibroblasts were cultivated in galactose medium or in standard glucose medium, and mitochondria were subsequently enriched by differential centrifugation and subjected to proteomic analysis. The purpose of galactose cultivation was to investigate the effect of metabolic stress and at the same time estimate analytical sensitivity and reproducibility of the proteomic method. The first step in the process of achieving mitochondrial proteome data was to enrich mitochondria. This organelle enrichment was instrumental since it decreased the complexity of the samples, and thus favored mass spectrometry detection of the mitochondrial proteins. The enrichment by differential centrifugation was relatively quick and was thus likely to be gentle to the mitochondria and their proteins. By assessing the enrichment factor an estimate of the amount of mitochondrial proteins in the cell was obtained. For this purpose an initial iTRAQ experiment was carried out, where the levels of mitochondrial proteins in enriched mitochondria were compared with those in total cell extracts (Figure 1). We detected 39 mitochondrial proteins with quantitative iTRAQ-ratios in all analyzed samples, and the average enrichment factor was 9.9 (SEM = 0.5) and 9.8 (SEM = 0.5) for cultivation in glucose and galactose, respectively [see Additional file 5].

The fibroblasts exhibited decreased growth rate in galactose medium; with approximately fifty percent longer generation time (data not shown). Since the mitochondria from galactose and glucose cultivation had similar enrichment ratios, it indicates that the slow growth rate in the cultures with galactose was not due to altered mitochondrial protein amount. Subsequent studies were conducted to explore the protein profiles of individual proteins sorted into mitochondrial pathways.

### Definition of mitochondrial pathways

Many mitochondrial pathways have been thoroughly studied and the gene products to a large extent are known. In order to facilitate interpretation of the proteome data we divided the main mitochondrial activities into nine different categories and defined the proteins belonging to the respective categories [see Additional file 6]. In addition to four metabolic categories there are three categories related to stress response, one to mitochondrial translation and one contains miscellaneous proteins. A criterion for inclusion into the sorted lists was that the proteins had previously been demonstrated or predicted to be localized to mitochondria [11]. For energy metabolism the pathways of the KEGG database [26,27] were adopted as an initial framework and manually curated using data from Gene Ontology [28] and literature data. The metabolic pathways are fatty acid oxidation (FAO), tricarboxylic (or citric) acid cycle (TCA) merged with pyruvate dehydrogenase (PDH), respiratory chain (RESP) and amino acid metabolism (AA). The other categories were primarily built bottom-up from literature data. The antioxidant systems (ANTIOX) category is composed of proteins that protect against oxidative stress; the translation (TRANS) category contains proteins of the mitochondrial translation machinery and protein quality control (PQC) category lists molecular chaperones and proteases. The apoptosis and mitochondrial morphology (APOP) category is composed of proteins known to play an important role for mitochondrial stress response and for influencing apoptosis regulation.

### Robust protein profile data

**Figure 2:**
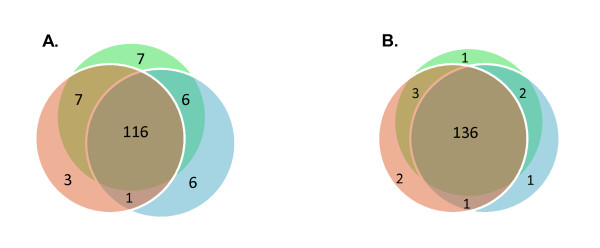
**Venn diagram showing the number of proteins with quantitative data in one, two or all three triplicate analyses**. A) Standard MS-setting and B) after addition of MS runs with 30 proteins targeted by SIM-analysis.

The mitochondrial protein samples were digested and labeled with iTRAQ reagent to obtain quantitative protein profiles comparing the two metabolic states. Protein identifications with low signal intensities might result in false positives and high analytical variance, and strict criteria were therefore applied to filter out such protein hits. First, three independent cultivation studies were performed followed by separate proteomic analyses. Second, only proteins that passed strict MS data criteria in all three experiments were included (see materials and methods). The statistical criteria on the MS data comprised both the protein identification probability and the peptide quantification procedure. Altogether, high quality data were obtained for relative quantification of more than one hundred mitochondrial proteins belonging to the pathways initially defined. For additional 30 proteins the initial experiments had quantitative data in only one or two of the three studies. To obtain triplicate values and to improve ion statistics, samples were subsequently re-analyzed on the MS using Selected Ion Monitoring (SIM), by which those 30 proteins were preferentially measured. Two peptides of each protein were put on an inclusion list and the samples were re-analyzed with the SIM MS-method. Following reanalysis, 136 instead of 116 proteins had sufficient data for inclusion into the dataset of mitochondrial proteins (Figure 2).

### Galactose versus glucose

**Figure 3:**
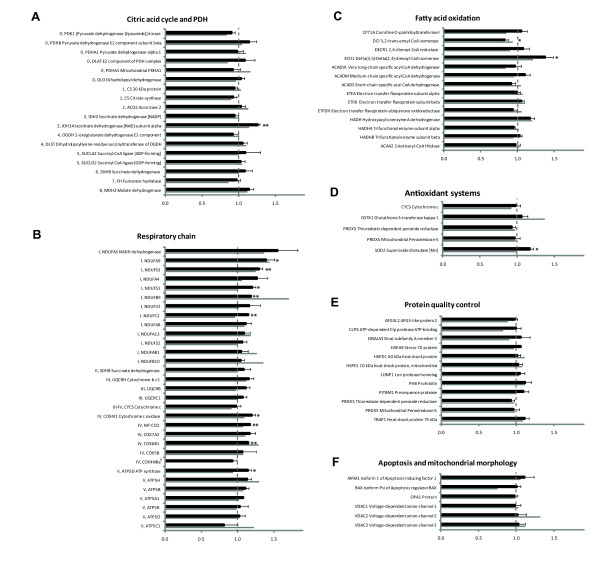
**Profiles of proteins belonging to six categories of mitochondrial pathways**. The pathways are: A) Citric acid cycle together with pyruvate dehydrogenase (PDH), B) Respiratory chain, C) Fatty acid oxidation, D) Antioxidant systems, E) Protein quality control and F) Apoptosis and mitochondrial morphology. The galactose to glucose ratio, derived from the protein levels of the cells cultivated in galactose and glucose, respectively, is depicted on the x-axis. Black bars indicate ratios calculated from the average of three independent cultivation studies of a control fibroblast (NHDF-1). The grey bar indicates ratio from one cultivation study of a second control fibroblast (NHDF-2). The ratio of a protein was reported as significantly different from 1.0 if it passed two tests 1) a threshold test of two times the global standard error (2 × 0.055 = 0.11) and 2) a two-tailed student's T-test for equal variance data. The error bar is the standard error of three values and "*" and "**" indicate statistically significant deviation from 1.0 with a t-test probability value below 0.05 and 0.01, respectively. A few proteins have activity in two pathways and are then depicted two times. The proteins in pathway A, B and C were sorted according to their position in the pathway, whereas the proteins in pathway D, E and F were alphabetically sorted based on the HGNC symbol, i.e. the letters that precedes the protein description.

A fibroblast line (NHDF-1) was cultivated on three occasions in galactose and glucose and the mitochondrial protein profiles were compared. Figure 3 and Additional file 7 contains galactose to glucose ratios of the identified proteins belonging to the defined functional categories. Several respiratory chain proteins exhibited increased levels as a function of galactose cultivation. The 13 detected complex I proteins had between 4 and 55% increased levels and for five of them the increase was statistically significant (Figure 3B). Proteins from all five respiratory chain complexes were detected and three proteins of complex IV and one of complex V also had significantly increased levels. In other pathways the ratios were close to unity for the majority of the proteins indicating that galactose did not significantly distort those pathways [see Additional file 7]. Single proteins with altered levels in galactose grown cells were NAD-dependent isocitrate dehydrogenase (HGNC symbol *IDH3A*) of the TCA cycle (26% increment, p < 0.01) and dienoyl-CoA isomerase (*ECH1*) of FAO (37% increment, p < 0.05). The other enzymes of TCA and FAO were only mildly influenced in response to galactose (Figure 3A and 3C).

To assess the reproducibility an additional fibroblast cell line (referred to as NHDF-2) was cultivated and analyzed using the same proteomic method. All of the 136 previously detected proteins were successfully detected, and only three of them had insufficient signal for quantification. Moreover, 13 of the 16 proteins with statistically significant change in the first study exhibited change in the same direction in the second study. Two of the three proteins that could not be confirmed (NDUFC2 and ATP5D) deviated in the opposite direction only weakly (less than six percent deviation from 1.0). For the third protein (OXCT1) no data was obtained due to insufficient quantification signal.

**Figure 4:**
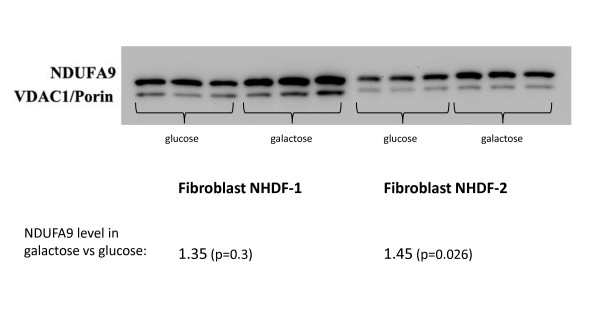
**Western blot detection of NDUFA9 and the loading control VDAC1 (porin)**. The samples were loaded in triplicate (6 μg protein/well) and the protein levels, NDUFA9 in relation to VDAC1, were compared for glucose versus galactose cultivation, for two different normal fibroblasts; NHDF-1 and NHDF-2.

The MS based relative quantification of the complex I component NDUFA9 consistently resulted in more than 35% elevation during galactose cultivation, although some of the other proteins of complex I were not elevated. To further confirm the NDUFA9 data we performed Western blot analysis and densitometry on samples from NHDF-1 and NHDF-2 (Figure 4). According to the data from this analysis the protein level of NDUFA9 was elevated 35% (p = 0.3) and 45% (p < 0.05) for NHDF-1 and NHDF-2, respectively, corroborating the MS analysis.

### Diseases related to mitochondrial function

Many mitochondrial proteins are involved in human diseases and their corresponding genes have been listed in the OMIM database of human diseases [29]. Additional file 8 shows the 38 gene products identified in the present study also described in OMIM. The detected proteins are involved in a wide range of diseases; spastic paraplegia (*HSPD1*), cancer (*BAX, PHB*), optic atrophy (*OPA1*), ethylmalonic encephalopathy (ETHE1) and Parkinson's disease (*NDUFV2*). The majority of the proteins are involved in metabolic diseases [see Additional file 8]. Moreover, a couple of the proteins linked to disease, NDUSF3 and NDUFS1 of respiratory complex I, were found to be galactose regulated.

## Discussion

Cultivated fibroblasts are commonly used for analyzing mitochondrial enzymatic activity and for detection of respiratory chain defects. In this study we applied proteomics for relative quantification of mitochondrial proteins and managed to cover the main pathways of mitochondrial activity obtaining in-depth data on energy metabolism and stress response. We analyzed the response of fibroblast cells to galactose cultivation and found that galactose resulted in increased levels of respiratory chain proteins. Respiration is crucial for growth in the slowly metabolized galactose [30] and fibroblast cells have previously been shown to be unable to grow in galactose when complex I or IV are defective [21,22]. Proteins of respiratory chain complex I, and to some extent complex IV, were in the present study found to be up-regulated in response to galactose. This might be a way for the cell to cope with the energy imbalance caused by galactose. Interestingly, a few proteins of complex I (e.g. NDUFA9, NDUFS3 and NDUFB9) were elevated to a higher degree than the rest, indicating sub-stoichiometric regulation of these proteins. Recently, it was shown that NDUFS3 was present in a matrix-soluble assembly and in several membrane-bound assemblies, including the holo-enzyme [31]. Further studies will have to elucidate whether the various complex I subunits are present in different subassemblies, which would explain the sub-stoichimetric relationship observed in the present study.

Protein profiles from the other functional categories displayed less difference between galactose and glucose cultivated cells. However, IDH3A, a part of the NAD^+ ^dependent isocitrate dehydrogenase (NADH-IDH) of the tricarboxylic acid cycle (TCA) was clearly increased when galactose was used as carbon source. NADH-IDH catalyzes the first NADH-yielding reaction of the TCA cycle, described to have a high impact of the overall rate of the TCA cycle and to be allosterically regulated, so that its activity can be increased in response to, for example, a low ATP to ADP ratio [32]. During galactose cultivation it seems that the allosteric activation was insufficient so that the protein amount of IDH3A enzyme also had to be increased, whereas the levels of the other TCA enzymes were close to unaffected. Diseases related to IDH3 are not described in the OMIM database. This could be related to the existence of the parallel and compensatory activity of NADP^+ ^-dependent IDH [33], which is able to compensate for the NADH-dependent activity of IDH3A. Recently, a loss-of-function of IDH3B, the β-subunit of NADH-IDH, was found in patients with retinitis pigmentosa. It is thus likely that deficient NADH-IDH activity is a contributory factor in energy deficiency disorders, causing severe symptoms only in certain tissues [33].

Single mitochondrial proteins might result in disease when they are present at an insufficient level, often caused by genetic variations resulting in protein misfolding and/or degradation [34]. Several of the proteins in Additional file 8 are associated with metabolic diseases, a group of diseases in which synergistic heterozygosity has been described, i.e. diseases resulting from multiple partial defects in one or more metabolic pathways [35,36]. For these diseases, the simultaneous quantification of several metabolic proteins is highly valuable for identification of the components of the synergistic effects. The present study detected 38 proteins related to disease according to the OMIM database. Since these disease-related proteins were detected in all of our experiments, their relative amounts are likely to be detectable in future, similar studies. Furthermore, there is growing evidence of links between metabolic defects, protein misfolding, oxidative stress, and disease [37]. It is therefore highly relevant to obtain protein profiles from multiple, well-defined pathways of metabolism and stress response to be able to study diseases involving multiple components.

## Conclusion

Quantitative mitochondrial proteomics of cultivated patient fibroblasts show promising results for exploring the consequences of genetic diseases at the protein level. Moreover, this type of method is suitable for environmental stress studies on cultivated human cells, as exemplified here by metabolic stress. Mapping of the interplay between various proteins and pathways might serve as a powerful tool for elucidation of the effects of disease and cellular stress. It was shown that when the cells had limited access to energy sources through cultivation in galactose, the amount of mitochondria did not seem to change; instead, the cells up-regulated parts of their respiratory pathway and specific metabolic proteins to compensate for the compromised energy state.

## Competing interests

The authors declare that they have no competing interests.

## Authors' contributions

NG and PB supervised and participated in the design of the study. JP, SV, CBP, PB and NG participated in definition and curation of pathway lists. VS and JP performed the laboratory experiments. JHC, VS and JP optimized the protocol for relative quantification. JP participated in the design of the study, performed data analysis and wrote the manuscript. All authors read and approved the final manuscript.

## Supplementary Material

Additional file 1 **Experimental protein identification data from the first experiment of fibroblast NHDF-1**. Mass spectrometry data including peptide list. Click here for file

Additional file 2 **Experimental protein identification data from the second experiment of fibroblast NHDF-1**. Mass spectrometry data including peptide list. Click here for file

Additional file 3 **Experimental protein identification data from the third experiment of fibroblast NHDF-1**. Mass spectrometry data including peptide list. Click here for file

Additional file 4 **Experimental protein identification data from the experiment of fibroblast NHDF-2**. Mass spectrometry data including peptide list. Click here for file

Additional file 5 **Enrichment factors of 39 mitochondrial proteins**. Enrichment factors of 39 mitochondrial proteins detected in an iTRAQ experiment, where a mitochondrial sample is compared with a cell sample, for human fibroblasts cultivated in glucose and galactose, respectively. The enrichment factor is calculated, for each protein, as the iTRAQ signal ratio: Mitochondrial sample/Total cell sample. Click here for file

Additional file 6 **Mitochondrial proteins sorted into functional categories**. The protein descriptions and isoform data are from the curated human IPI database  version 3.45 with 71,983 sequences, released 6/10/2008. All the sorted proteins have been predicted to be mitochondrial according to Pagliarini *et al *[11], and the total list of those mitochondrial proteins is below the sorted categories (starting at row 978). The categories are: Fatty acid metabolism, Citric acid cycle, Amino acid metabolism, Respiratory chain, Protein quality control systems, Antioxidant systems, Mitochondrial morphology, Mitochondrial translation and Apoptosis. Click here for file

Additional file 7 **Protein profiles of mitochondrial amino acid metabolism (AA), mitochondrial translation (TRANS) and miscellaneous mitochondrial proteins (MISC)**. The galactose to glucose ratio, derived from the protein levels of the cells cultivated in galactose and glucose, respectively, is depicted on the x-axis. Black bars indicate ratios calculated from the average of three independent cultivation studies of a control fibroblast (NHDF-1). The grey bar indicates the ratio from one cultivation study of a second control fibroblast (NHDF-2). A ratio of a protein was reported as significantly different from 1.0 if it passed two tests 1) a threshold test of two times the global standard error (2 × 0.055 = 0.11) and 2) a two-tailed student's T-test for equal variance data. The error bar is the standard error of the three values and "*" and "**" indicate statistically significant deviation from 1.0 with t-test probability value below 0.05 and 0.01, respectively. Click here for file

Additional file 8 **Gene products detected in the present study which have known disease association according to the OMIM database**. Abbreviations: amino acid metabolism (AA), apoptosis and mitochondrial morphology (APOP), citric acid cycle (TCA), fatty acid oxidation (FAO), miscellaneous (MISC), protein quality control (PQC), respiratory chain (RESP) and translation (TRANS). Click here for file

## References

[B1] Chan DC (2006). Mitochondria: dynamic organelles in disease, aging, and development. Cell.

[B2] Janssen RJ, Heuvel LP van den, Smeitink JA (2004). Genetic defects in the oxidative phosphorylation (OXPHOS) system. Expert Rev Mol Diagn.

[B3] Gregersen N, Bross P, Andresen BS (2004). Genetic defects in fatty acid beta-oxidation and acyl-CoA dehydrogenases. Molecular pathogenesis and genotype-phenotype relationships. Eur J Biochem.

[B4] Kwong JQ, Beal MF, Manfredi G (2006). The role of mitochondria in inherited neurodegenerative diseases. J Neurochem.

[B5] Del Poeta G, Bruno A, Del Principe MI, Venditti A, Maurillo L, Buccisano F, Stasi R, Neri B, Luciano F, Siniscalchi A, de Fabritiis P, Amadori S (2008). Deregulation of the mitochondrial apoptotic machinery and development of molecular targeted drugs in acute myeloid leukemia. Curr Cancer Drug Targets.

[B6] Kroemer G, Pouyssegur J (2008). Tumor cell metabolism: cancer's Achilles' heel. Cancer Cell.

[B7] White MY, Edwards AVG, SJ C, Van Eyk JE (2008). Mitochondria: A mirror into cellular dysfunction in heart disease. Proteomics Clin Appl.

[B8] Jüllig M, Hickey AJ, Middleditch MJ, Crossman DJ, Lee SC, Cooper GJS (2007). Characterization of proteomic changes in cardiac mitochondria in streptozotocin-diabetic rats using iTRAQ™ isobaric tags. Proteomics Clin Appl.

[B9] Lin MT, Beal MF (2006). Mitochondrial dysfunction and oxidative stress in neurodegenerative diseases. Nature.

[B10] Calvo S, Jain M, Xie X, Sheth SA, Chang B, Goldberger OA, Spinazzola A, Zeviani M, Carr SA, Mootha VK (2006). Systematic identification of human mitochondrial disease genes through integrative genomics. Nat Genet.

[B11] Pagliarini DJ, Calvo SE, Chang B, Sheth SA, Vafai SB, Ong SE, Walford GA, Sugiana C, Boneh A, Chen WK, Hill DE, Vidal M, Evans JG, Thorburn DR, Carr SA, Mootha VK (2008). A mitochondrial protein compendium elucidates complex I disease biology. Cell.

[B12] Taylor SW, Fahy E, Zhang B, Glenn GM, Warnock DE, Wiley S, Murphy AN, Gaucher SP, Capaldi RA, Gibson BW, Ghosh SS (2003). Characterization of the human heart mitochondrial proteome. Nat Biotechnol.

[B13] Gaucher SP, Taylor SW, Fahy E, Zhang B, Warnock DE, Ghosh SS, Gibson BW (2004). Expanded coverage of the human heart mitochondrial proteome using multidimensional liquid chromatography coupled with tandem mass spectrometry. J Proteome Res.

[B14] Zhang J, Li X, Mueller M, Wang Y, Zong C, Deng N, Vondriska TM, Liem DA, Yang JI, Korge P, Honda H, Weiss JN, Apweiler R, Ping P (2008). Systematic characterization of the murine mitochondrial proteome using functionally validated cardiac mitochondria. Proteomics.

[B15] Gloerich J, Wevers RA, Smeitink JA, van Engelen BG, Heuvel LP van den (2007). Proteomics approaches to study genetic and metabolic disorders. J Proteome Res.

[B16] Ross PL, Huang YN, Marchese JN, Williamson B, Parker K, Hattan S, Khainovski N, Pillai S, Dey S, Daniels S, Purkayastha S, Juhasz P, Martin S, Bartlet-Jones M, He F, Jacobson A, Pappin DJ (2004). Multiplexed protein quantitation in Saccharomyces cerevisiae using amine-reactive isobaric tagging reagents. Mol Cell Proteomics.

[B17] Wu WW, Wang G, Baek SJ, Shen RF (2006). Comparative study of three proteomic quantitative methods, DIGE, cICAT, and iTRAQ, using 2D gel- or LC-MALDI TOF/TOF. J Proteome Res.

[B18] Bantscheff M, Boesche M, Eberhard D, Matthieson T, Sweetman G, Kuster B (2008). Robust and sensitive iTRAQ quantification on an LTQ Orbitrap mass spectrometer. Mol Cell Proteomics.

[B19] Bantscheff M, Schirle M, Sweetman G, Rick J, Kuster B (2007). Quantitative mass spectrometry in proteomics: a critical review. Anal Bioanal Chem.

[B20] Ernoult E, Gamelin E, Guette C (2008). Improved proteome coverage by using iTRAQ labelling and peptide OFFGEL fractionation. Proteome Sci.

[B21] Robinson BH, Petrova-Benedict R, Buncic JR, Wallace DC (1992). Nonviability of cells with oxidative defects in galactose medium: a screening test for affected patient fibroblasts. Biochem Med Metab Biol.

[B22] Hofhaus G, Johns DR, Hurko O, Attardi G, Chomyn A (1996). Respiration and growth defects in transmitochondrial cell lines carrying the 11778 mutation associated with Leber's hereditary optic neuropathy. J Biol Chem.

[B23] Lengqvist J, Uhlen K, Lehtio J (2007). iTRAQ compatibility of peptide immobilized pH gradient isoelectric focusing. Proteomics.

[B24] Olsen JV, de Godoy LM, Li G, Macek B, Mortensen P, Pesch R, Makarov A, Lange O, Horning S, Mann M (2005). Parts per million mass accuracy on an Orbitrap mass spectrometer via lock mass injection into a C-trap. Mol Cell Proteomics.

[B25] Westhuizen FH van der, Heuvel LP van den, Smeets R, Veltman JA, Pfundt R, van Kessel AG, Ursing BM, Smeitink JA (2003). Human mitochondrial complex I deficiency: investigating transcriptional responses by microarray. Neuropediatrics.

[B26] Kanehisa M, Goto S (2000). KEGG: kyoto encyclopedia of genes and genomes. Nucleic Acids Res.

[B27] Kanehisa M, Araki M, Goto S, Hattori M, Hirakawa M, Itoh M, Katayama T, Kawashima S, Okuda S, Tokimatsu T, Yamanishi Y (2008). KEGG for linking genomes to life and the environment. Nucleic Acids Res.

[B28] Ashburner M, Ball CA, Blake JA, Botstein D, Butler H, Cherry JM, Davis AP, Dolinski K, Dwight SS, Eppig JT, Harris MA, Hill DP, Issel-Tarver L, Kasarskis A, Lewis S, Matese JC, Richardson JE, Ringwald M, Rubin GM, Sherlock G (2000). Gene ontology: tool for the unification of biology. The Gene Ontology Consortium. Nat Genet.

[B29] McKusick-Nathans Institute of Genetic Medicine JHUB, MD) and National Center for Biotechnology Information, National Library of Medicine (Bethesda, MD) (2008). Online Mendelian Inheritance in Man, OMIM (TM). http://www.ncbi.nlm.nih.gov/sites/entrez?db=OMIM.

[B30] Rossignol R, Gilkerson R, Aggeler R, Yamagata K, Remington SJ, Capaldi RA (2004). Energy substrate modulates mitochondrial structure and oxidative capacity in cancer cells. Cancer Res.

[B31] Dieteren CE, Willems PH, Vogel RO, Swarts HG, Fransen J, Roepman R, Crienen G, Smeitink JA, Nijtmans LG, Koopman WJ (2008). Subunits of mitochondrial complex I exist as part of matrix- and membrane-associated subcomplexes in living cells. J Biol Chem.

[B32] Gabriel JL, Zervos PR, Plaut GW (1986). Activity of purified NAD-specific isocitrate dehydrogenase at modulator and substrate concentrations approximating conditions in mitochondria. Metabolism.

[B33] Hartong DT, Dange M, McGee TL, Berson EL, Dryja TP, Colman RF (2008). Insights from retinitis pigmentosa into the roles of isocitrate dehydrogenases in the Krebs cycle. Nat Genet.

[B34] Gregersen N, Bolund L, Bross P (2005). Protein misfolding, aggregation, and degradation in disease. Mol Biotechnol.

[B35] Vockley J, Rinaldo P, Bennett MJ, Matern D, Vladutiu GD (2000). Synergistic heterozygosity: disease resulting from multiple partial defects in one or more metabolic pathways. Mol Genet Metab.

[B36] Schuler AM, Gower BA, Matern D, Rinaldo P, Vockley J, Wood PA (2005). Synergistic heterozygosity in mice with inherited enzyme deficiencies of mitochondrial fatty acid beta-oxidation. Mol Genet Metab.

[B37] Gregersen N, Bross P, Vang S, Christensen JH (2006). Protein Misfolding and Human Disease. Annu Rev Genomics Hum Genet.

