# Supplementary figures and images for: Mitochondrial proteomics on human fibroblasts for identification of metabolic imbalance and cellular stress
Source: Proteome Sci. 2009 May 28;7:20. doi: 10.1186/1477-5956-7-20 (PMC2695441; doi:10.1186/1477-5956-7-20)

### Amino acid metabolism

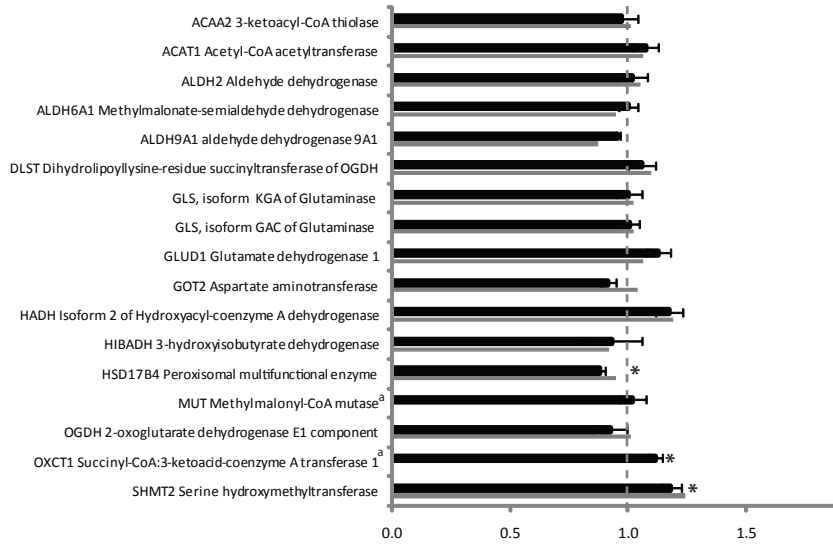

### Translation

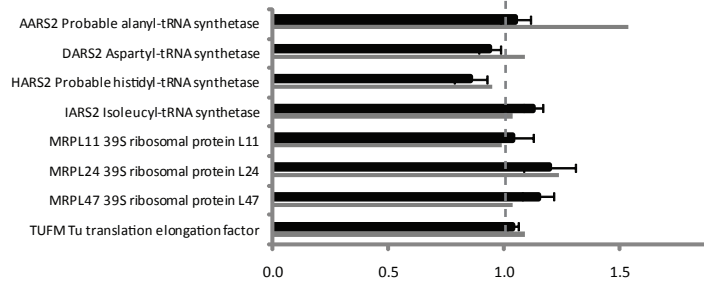

### Miscellaneous

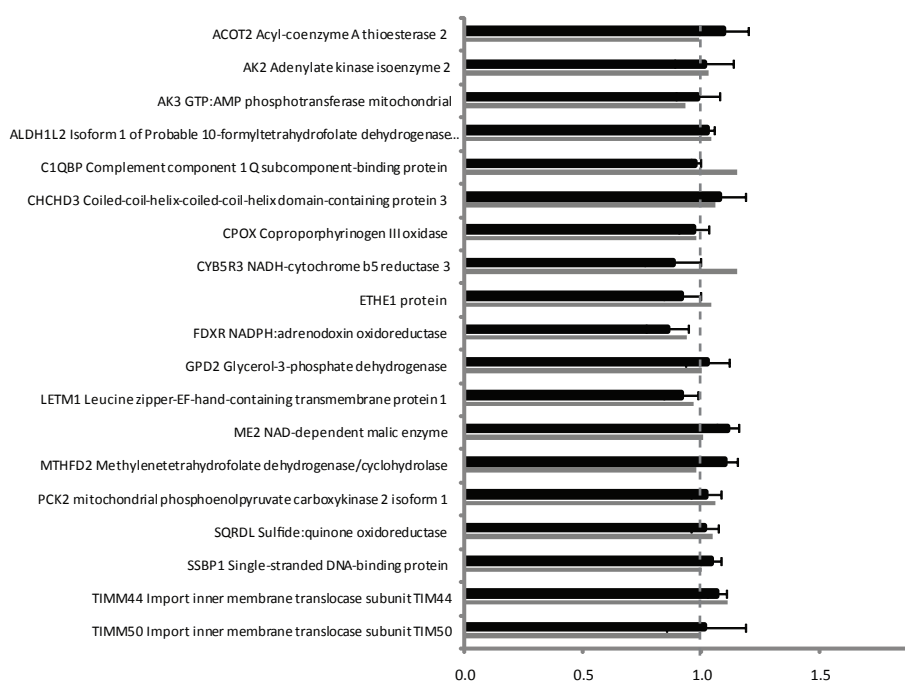

Supplement: Additional file 7 — Protein profiles of mitochondrial amino acid metabolism (AA), mitochondrial translation (TRANS) and miscellaneous mitochondrial proteins (MISC). The galactose to glucose ratio, derived from the protein levels of the cells cultivated in galactose and glucose, respectively, is depicted on the x-axis. Black bars indicate ratios calculated from the average of three independent cultivation studies of a control fibroblast (NHDF-1). The grey bar indicates the ratio from one cultivation study of a second control fibroblast (NHDF-2). A ratio of a protein was reported as significantly different from 1.0 if it passed two tests 1) a threshold test of two times the global standard error (2 × 0.055 = 0.11) and 2) a two-tailed student's T-test for equal variance data. The error bar is the standard error of the three values and "*" and "**" indicate statistically significant deviation from 1.0 with t-test probability value below 0.05 and 0.01, respectively. [file 1477-5956-7-20-S7.pdf]
