# Supplementary material for: Mitochondrial proteomics on human fibroblasts for identification of metabolic imbalance and cellular stress
Source: Proteome Sci. 2009 May 28;7:20. doi: 10.1186/1477-5956-7-20 (PMC2695441; doi:10.1186/1477-5956-7-20)
Supplement: Additional file 8 — Gene products detected in the present study which have known disease association according to the OMIM database. Abbreviations: amino acid metabolism (AA), apoptosis and mitochondrial morphology (APOP), citric acid cycle (TCA), fatty acid oxidation (FAO), miscellaneous (MISC), protein quality control (PQC), respiratory chain (RESP) and translation (TRANS). [file 1477-5956-7-20-S8.pdf]

HGNC gene code	Pathway	Protein	Disease	OMIM (gene)	OMIM (disease)
ACADM	FAO	Medium-chain Acyl-CoA dehydrogenase (MCAD)	MCAD deficiency	607008	201450
ACADVL	FAO	Very long-chain Acyl-CoA dehydrogenase (VLCAD)	VLCAD deficiency	609575	201475
ACAT1	AA	Acetoacetyl-CoA thiolase	β-ketothiolase deficiency	607809	203750
ALDH2	MISC	Aldehyde dehydrogenase 2	Alcohol sensitivity, handover	100650	610251
ALDH6A1	AA	Methylmalonate semialdehyde dehydrogenase (MMSD)	MMSD deficiency	603178	603178
BAX	APOP	Bcl2 associated protein x	Cancer susceptibility	600040	600040
CPT1A	FAO	Hepatisk CPT1	Hepatisk CPT1 deficiency	600528	255120
DARS2	TRANS	Aspertyl t-RNA synthetase	Leukoencephalopathy	610956	611105
DECR1	MISC	2,4-dienoyl-CoA reductase (DECR)	DECR deficiency	222745	222745
DLD	TCA	Dihydrolipoamid dehydrogenase E3 subunit	α-keto glutarate dehydrogenase deficiency	238331	238331
ETFA	FAO	Electron transfer flavoprotein (ETF), α subunit	Multiple acyl-CoA dehydroganase deficiency (MADD), Glutaric aciduria, type II (GAII)	608053	231680
ETFB	FAO	Electron transfer flavoprotein (ETF), β subunit	Multiple acyl-CoA dehydroganase deficiency (MADD), Glutaric aciduria, type II (GAII)	130410	231680
ETFDH	FAO	Electron transfer flavoprotein ubiquinine oxidoreductase, ETFQO	Multiple acyl-CoA dehydroganase deficiency (MADD), Glutaric aciduria, type II (GAII)	231675	231680
ETHE1	MISC	ETHE1 protein	Ethylmalonic encephalopathy	608451	602473
FH	TCA	Fumarase	Fumarase deficiency	136850	606812
GLUD1	AA	Glutamate dehydrogenase 1	Hyperinsulinism-hyperammonemia syndrome	138130	606762
GPD2	MISC	Glycerol 3-phosphate dehydrogenase 2	Noninsulin dependent diabetes mellitus	138430	125853
HADH	FAO	3-hydroxyacyl-CoA dehydrogenase, HADH (or M/SCHAD)	HADH (or M/SCHAD) deficiency	601609	231530
HADHA	FAO	Mitochondrial trifunctional protein (MTP), α subunit	LCHAD deficiency; MTP deficiency	600890	609016
HADHB	FAO	Mitochondrial trifunctional protein (MTP), β subunit	MTP deficiency	143450	609015
HSD17B4	MISC	17-β hydroxy steroid dehdrogenase IV, D-bifinctional protein	D-bifunctional protein deficiency	601860	261515
HSPD1	PQC	Heat shock 60-kd protein (HSP60)	Spastic paraplegia 13	118190	605280
ME2	MISC	Malic enzyme 2	Susceptibility to idopathic generalized epilepsy	154270	600669
NDUFA13	RESP	NADH-ubiquinone oxidoreductase 1α	Papillary thyroid carcinoma	609435	607464
[a]*NDUFS1*	RESP	NADH-ubiquinone oxidoreductase Fe-S 1	Mitochondrial complex I deficiency	157655	252010
NDUFS2	RESP	NADH-ubiquinone oxidoreductase Fe-S 2	Mitochondrial complex I deficiency	602985	252010
[a]*NDUFS3*	RESP	NADH-ubiquinone oxidoreductase Fe-S 3	Mitochondrial complex I deficiency	603846	256000
NDUFV2	RESP	NADH-ubiquinone oxidoreductase flavoprotein 2	Mitochondrial complex I deficiency	600532	168600
OGDH	TCA	α-keto glutarate dehydrogenase (E1k)	α-keto glutarate dehydrogenase deficiency	203740	-
OPA1	APOP	Optic atrophy 1 protein	Mitochondrial dysfunction	605290	165500
PCK2	MISC	Phosphorenolpyruval carboxykinase	PEPCK deficiency, mitochondrial	261650	-
PDHA1	TCA	Pyruvate dehydrogenase complex E1α	Pyruvate dehydrogenase deficiency	312170	308930
PDHB	TCA	Pyruvate dehydrogenase complex E1β	Pyruvate dehydrogenase deficiency	312170	179060
PHB	PQC	Prohibitin	Breast cancer, sporadic	176705	-
SDHB	TCA / RESP	Succinate dehydrogenase subunit B	Paraganglioma and gastric stromal sarcoma	185470	606864
SUCLA2	TCA	Succinate-CoA ligase subunit β	Mitochondrial DNA depletion syndrome,	603921	612073

[a] Gene products with statistically significant altered level in the galactose cultivations.
